# Infants and young children feeding practices and nutritional status in two districts of Zambia

**DOI:** 10.1186/s13006-015-0033-x

**Published:** 2015-02-18

**Authors:** Mary Katepa-Bwalya, Victor Mukonka, Chipepo Kankasa, Freddie Masaninga, Olusegun Babaniyi, Seter Siziya

**Affiliations:** World Health Organization, Country Office, Lusaka, Zambia; Public Health Unit, Clinical Sciences Department, School of Medicine, Copperbelt University, Ndola, Zambia; Department of Paediatrics and Child Health, University Teaching Hospital, Lusaka, Zambia

**Keywords:** Exclusive breastfeeding, Infant feeding practices, Nutritional status

## Abstract

**Background:**

Appropriate feeding is important in improving nutrition and child survival. Documentation of knowledge of caregiver on infant feeding is scanty in Zambia. The aim of this study was to describe feeding practices and nutritional status among infants and young children (IYC) in two districts in Zambia: Kafue and Mazabuka.

**Methods:**

A cross-sectional study was conducted between January and March 2006 using both quantitative and qualitative methods. A questionnaire was administered to caregiver of children aged under24 months. Lengths and weights of all children were measured. Focused group discussions were conducted in selected communities to assess parents or guardian knowledge, attitude and practice related to infant feeding.

**Results:**

A total of 634 caregivers (361 from Kafue and 273 from Mazabuka) participated in the study. About 311/618 (54.0%) of the caregiver knew the definition and recommended duration of exclusive breastfeeding (EBF) and when to introduce complementary feeds. Two hundred and fifty-one (81.2%) out of 310 respondents had acquired this knowledge from the health workers. Only 145/481 (30.1%) of the respondents practiced exclusive breastfeeding up to six months with 56/626 (8.9%) of the mothers giving prelacteal feeds. Although 596/629 (94.8%) of the respondents reported that the child does not need anything other than breast milk in the first three days of life, only 318/630 (50.5%) of them considered colostrum to be good. Complementary feeds were introduced early before six months of age and were usually not of adequate quality and quantity. Three hundred and ninety-one (64%) out of 603 caregivers knew that there would be no harm to the child if exclusively breastfed up to six months. Most of the children’s nutritional status was normal with 25/594 (4.2%) severely stunted, 10/596 (1.7%) severely underweight and 3/594 (0.5%) severely wasted.

**Conclusions:**

The caregiver in the communities knew about the recommended feeding practices, but this knowledge did not translate into good practice. Knowing that most of the mothers will breastfeed and have heard about appropriate breastfeeding, is important in the development of sustainable strategies required to improve feeding practices and, thus, nutritional status of children.

## Background

The Zambia Demographic and Health Surveys (ZDHS) have shown a declining trend in child mortality rates [1]. However, current rates remain unacceptably high, which necessitates concerted efforts to increase the coverage of known cost-effective and low-cost interventions to enable Zambia attain the Millennium Development Goal 4 (MDG 4) target of reducing child mortality from 167 per 1000 to 64 per 1000 live births. A key issue that needs to be addressed in trying to meet MDG 4 is under nutrition which still underlies almost a third of all deaths in children less than five years [2]. Estimates indicate about 35% of child death and 11% of total global disease burden are due to poor nutrition [3]. According to the 2002 Zambia Demographic and Health Survey, 42% of children dying had underlying malnutrition [4]. Appropriate feeding practices are fundamental to survival, growth, development, health and nutrition of infants and children and to the well-being of the mothers. There is sufficient evidence of cause effect for certain preventive interventions such as exclusive breastfeeding in the first six months in the prevention of diarrhoea, pneumonia and neonatal sepsis; complementary feeding in preventing diarrhoea, pneumonia, measles and malaria; and vitamin A in prevention of diarrhoea [5]. Interventions including breastfeeding, complementary feeding, vitamin A and zinc supplementation could save about 25% of total deaths in under-five age group [5]. Breastfeeding alone has been shown to decrease the child mortality by 13%. Promoting appropriate infant feeding practices such as early initiation of breastfeeding and exclusive breastfeeding for up to six months, even in the era of HIV and AIDS, is an effective strategy for improving child survival. Even though breastfeeding is universally practiced in Zambia, early initiation of breastfeeding occurs in only 57% of cases. Nine percent of infants receive prelacteal feeds. The national median duration of exclusive breastfeeding is three months. This shows that a significant number of infants still do not benefit from optimum breastfeeding practices. In sub-Saharan Africa, 31% of children are exclusively breastfed for up to six months [6]. Infants aged zero to five months who are not breastfed have a higher risk of dying as compared to their counterparts who are breastfed [7-9]. Exclusive breastfeeding has been shown to decrease the rates of HIV (Human immunodeficiency virus) transmission as compared to mixed feeding [10]. In Africa most women breastfeed their children for up to two years of age, but seldom practice WHO (World Health Organization) recommended exclusive breastfeeding up to six months of age [11-13]. This is in part due to lack of knowledge on the protective effects of exclusive breastfeeding for the recommended duration [14]. The zero to five months exclusive breastfeeding rates for Zambia has steadily increased from 13 to 61% in 1992 and 2007 respectively [1]. The median duration of breastfeeding in Zambia is reported to be 21 months. This is below the WHO/UNICEF optimum recommendation of breastfeeding for two years and above.

Information on parents or guardians knowledge and practice on infant feeding is limited in Zambia. This study aimed to describe feeding practices and nutritional status among infants and young children (IYC) in two districts in Zambia, Kafue and Mazabuka. The information gathered seeks to strengthen delivery of infant and young child feeding (IYCF) interventions and thus improve nutrition status and child survival.

## Methods

### Study design and data collection

This was a cross-sectional study that combined qualitative and quantitative methods. Qualitative data on infant feeding practices was obtained through focus group discussions (FGD), semi-structured interviews and key informant interviews in Mazabuka.

For the quantitative part of the study, a questionnaire was administered to the caregiver by 22 trained research assistants recruited from Kafue and Mazabuka. The questionnaire administered to caregiver collected information on the general characteristics of the participants and their children, knowledge, attitude and practices on early initiation of breastfeeding, colostrum, exclusive breastfeeding for up to six months, introduction of complementary foods and types of complementary feeds, breastfeeding problems and how they are addressed and support for breastfeeding. The weight and length of the children were measured using a salter scale and length board respectively. Research assistants were trained over a two day period and the quantitative study was conducted between January and March 2006.

### Study sample

The mothers and their infants or toddlers were the primary targets. If the mother was not at home at the time of the survey, the questionnaire was administered to the closest guardian of children aged zero to twenty three months who was at home.

Nine FGD were conducted in Mazabuka among women who were younger than 25 years and women older than 25 years. Eighteen in depth interviews with fathers, grandmothers, health workers and traditional birth attendants were conducted.

### Sample size and sampling

The sample size was determined using the 2002 Zambia Demographic and Health Survey exclusive breastfeeding prevalence rate of 40% [4] at a confidence level of 95%. Considering a response rate of 90%, the minimum required sample size was estimated as 450.

### Sampling

In urban areas participants were randomly selected. However, in the rural areas, a convenient sample was collected because the rural households were scattered over large hard to reach geographical areas.

### Study site

The study was conducted in two settlements (urban and rural) in Mazabuka district and similarly, two settlements in Kafue districts. Baseline data was being collected in Mazabuka for an intervention study that was to be implemented the following year. Kafue was conveniently added as it is a district near Mazabuka.

### Data management and analysis

The Principal Investigator supervised the data collection and management. Two data entry clerks entered the same data simultaneously. Microsoft Excel and SPSS were used for data entry and analysis respectively. Quality control audits of all data in the database were made daily after entering data from each field exercise.

Primary outcome variables were breastfeeding rates, exclusive breastfeeding rate and duration of breastfeeding; and secondary outcome variables were caregiver’ knowledge of complementary feeding. The Chi square test was used to compare proportions between districts at the 5% significance level.

### Ethics

The study received clearance from the University of Zambia Research Ethics Committee and the Ministry of Health through the district health management teams (DHMT) in Kafue and Mazabuka. Individual informed written consent was obtained from all participants of the study before any instrument was administered to them.

## Results

All caregiver in the selected households consented to participating in the survey. A total of 634 (273 from Mazabuka and 361 from Kafue) caregiver participated in the study. The questionnaire was administered to all identified caregiver residing in the selected households. 621/630 (98.6%) of the caregivers were the mothers of the children. Educational level of the parents/guardian was significantly (p < 0.001) higher in Mazabuka than in Kafue (Table 1). About 365/629 (58.0%) of the children seen were infants.

**Table 1:** **Characteristics of the study population**

	**Mazabuka**		**Kafue**		
	**(Total = 273)**		**(Total = 361)**		
	**%**	**(n)**	**%**	**(n)**	**p-value**
**Sex of head of family**					
Male	84	(226)	92	(327)	0.003
Females	16	(43)	8	(30)	
**Age of caretaker (years)**					
<19	15	(41)	14	(51)	0.96
20-29	59	(161)	59	(212)	
30-39	22	(60)	23	(83)	
40-59	3.3	(9)	4	(14)	
**Caretaker-child relationship**					
Mother	98.2	(268)	99	(357)	0.368
Other	1.8	(5)	1	(4)	
**Marital status**					
Married	81.8	(220)	85	(308)	0.265
Widowed	4.1	(11)	2	(8)	
Divorced	3	(8)	2	(8)	
Separated	1.1	(3)	2	(8)	
Single	10	(27)	7	(25)	
**Educational level-caretaker**					
None	5.2	(14)	11	(38)	<0.001
Primary	50.6	(137)	67	(239)	
Secondary	38	(103)	23	(81)	
Tertiary	6.3	(17)	0	(1)	
**Age distribution of the children**					
<6 months	28	(76)	31	(111)	
6 – 11 months	26.2	(71)	30	(107)	
>12 months	45.8	(124)	39	(140)	

### Initiation of breastfeeding

Six hundred and twenty-one (99.4%) out of 625 mothers reported having put their infants to the breast. The majority of them reported to have started breastfeeding within the first hour after delivery. During focus group discussions most mothers stated that they breastfed their child within an hour after delivery. Their response was as follows: “*You breastfeed your child when you finish giving birth*”. In the FGD conducted in urban area mothers emphasized that the child needed to cry before the child is put to the breast. “*When I deliver, if my baby has not cried, then I don’t breastfeed. But if she/he cries, then that is when I breastfeed*”. Only 34/267 (12.7%) of the respondents in Mazabuka and 22/359 (6.1%) in Kafue (p = 0.004) gave prelacteal feeds. The respondents were taught information on the need to initiate breastfeeding in the first hour and the avoidance of prelacteal feeds during the focused antenatal clinic (FANC) and under five clinic (U5C) visits. FGD conducted in the rural areas some of the discussant said that there was no need to give anything before starting to breastfeed whereas others felt there was need to give a little water . . . “*I normally give water. It makes the throat wet. The throat should not be dry, because the milk at that time has not yet started coming out*”.

About half of mothers in both districts talked positively about colostrum. They indicated that it was good for the child, while about a third of the caregiver in both Mazabuka and Kafue thought that the colostrum was bad for the child. The rest of the mothers did not have an opinion about the colostrum. Respondents in the urban FGD talked about colostrum as “appearing like water” and not milk. They indicated that it was good and protected the baby from diseases. They had obtained information on colostrum from the elderly people in the community and the health workers during health talks given at antenatal clinic visits. In the rural areas, a few of the discussants reported squeezing out and discarding colostrum as it was thought to be dirty. They reported that colostrum was not milk but “*it brought in milk*”. Significantly more caregivers in Kafue (254/261, 97.5%) than in Mazabuka (317/348, 91.1%) reported that the child does not need anything other than breast milk in the first three days of life (p < 0.001).

### Exclusive breastfeeding

There was a significantly higher (p < 0.001) proportion of children who were exclusively breastfed up to four months in Kafue (207/301, 68.8%) than in Mazabuka (98/186, 53.0%). The rate of EBF dropped at age six months (56/181, 31.1%) in Mazabuka and (89/300, 29.9%) in Kafue). The FGD showed that mothers were generally aware of the concept of exclusive breastfeeding (EBF) and most of them had a positive perception about it. The main reason given in favour of EBF was that the intestines of the infant were too immature and small to handle other foods and the baby would get sick and have stomach pains if given other foods. About 391/606 (64%) of the caregiver in both districts knew that no harm would come to the child if he/she was exclusively breastfed up to six months and that the breast milk was enough for the child. Most of the discussants in the FGD reported that the baby can grow healthy on breast milk alone and can survive for six months. The main barrier to practicing EBF was that the caregiver feared that babies would not be accustomed to other foodstuff in case the mother is not able to breastfed due to illness or death. Some discussants indicated that mothers may not have adequate breast milk and would need to introduce other feeds early. For such problems, increasing the food intake and intake of locally brewed non-alcoholic maize mealie meal based drinks such as *‘munkoyo*’ and *‘chibwantu’* were considered to be plausible ways of increasing breast milk production. None of the discussants in the FGD discussed the risk of HIV infection related to mixed feeding or the importance of EBF in this era of HIV.

### Complementary feeds

316/486 (65.0%) caregivers knew that other foods should be introduced at six months. There were significantly more caregivers in Mazabuka as compared to Kafue who knew this (p < 0.001). Likewise, significantly more caregivers in Kafue (288/360, 80.0%) than in Mazabuka (178/255, 69.8%) reported that the child would show signs of being ready for other foods (p = 0.004). Some of the signs identified were crying a lot, an increase in appetite and wanting to breastfeed more often than usual. Other discussants felt that milk was a ‘liquid’ and not ‘food’ and the child needed ‘food’ to satisfy his/her hunger. The most commonly introduced complementary food at six months was maize mealie porridge. What was given in addition to the porridge was dependent on the economic status of the discussants. Addition of groundnuts, pounded small fish (*kapenta*), oil, sugar, egg (especially the yolk), bean soup, and milk to the maize meal porridge was common. FGD showed that caregivers residing in the urban areas who could afford, introduced a commercially pre-cooked cereal called ‘*cerelac*’ before the maize meal porridge. This was mentioned in both the urban and rural FGD, although the latter discussants said that they could not afford to buy the ‘*cerelac*’. Fruits, commonly bananas and oranges, were mentioned as being common complementary foods that were given to children.

### Continued breastfeeding

Eighty nine percent (558/627) of caregivers were breastfeeding their children at the time of the interviews. They all reported to be breastfeeding during the day and night. Duration of breastfeeding for those who had stopped breastfeeding ranged from one to twenty-two months. The majority stopped breastfeeding because they believed breast milk was not enough or that the child had lost interest in breastfeeding. The median age for stopping breastfeeding was 18 (Q_1_ = 16, Q_3_ = 20) months in both districts. The discussants in the FGD (both rural and urban) reported to have stopped breastfeeding their children between 18 and 24 months. The majority felt that at this age, the child does not need breast milk. Ways of determining if the child was old enough to be weaned included the following: child being able to walk and obey requests like bringing an item to the mother. In addition some children were weaned off if the mother became pregnant. A number of the discussants reported that they took the child to the grandparents when they wanted him/her to be weaned off breast milk. “W*hen a child wants to stop, you take him/her to someone’s place - Like at his grandparents place or if I have a ‘big’ [elder] sister or brother I can take him there”.* One discussant reported: “*No, like me, when I want to wean my child, I went to seek advice from the health centre on what I should give my baby. So, I was told to buy drinks”.* Drinks talked about were locally made maize mealie non-alcoholic based drinks and brews like *maheu, chibwantu* and *umunkoyo*.

### Support for breastfeeding

The father of the child was identified as being the main source of financial and moral support pertaining to infant feeding. Other sources of support identified were relatives like the grandparents and aunties. Some of the discussants felt that older people who had experience with looking after children could give valuable advice although some of the advice was incorrect. They indicated nurses to be an important source of support as they were very knowledgeable and shared their knowledge during the antenatal and under five clinic visits.

### Difficulties in breastfeeding

Of 617 respondents, 572 (92.7%) did not report any difficulties in breastfeeding. Of the few that reported difficulties, the common problems were pain when breastfeeding, infection, breast abscess and sore nipples. In addition to these difficulties, they identified lack of milk, or mother being too busy to breastfeed, or mother being sick from HIV or the breast milk being “bad”. When asked about whether a child should stop breastfeeding in the event of the mother falling sick, the majority in the FGD said that it depended on the type of illness. It was felt that the mother should stop breastfeeding if she had diseases like severe malaria, tuberculosis and acquired immune deficiency syndrome (AIDS). On the other hand, if the child was sick, the majority of the women felt that continuing breastfeeding was good as breast milk gave the baby strength and energy as well as protected the child and prevented the progression of the child’s illness. About one third of respondents who had difficulties whilst breastfeeding went to a health facility to seek help. When asked who they would consult if they had breastfeeding problem, those who did not have problems reported that they would seek help from a health worker (523/607, 86.2%) followed by the maternal grandmother (50/607, 8.2%). In addition to the health worker, discussants in the rural areas indicated the grandmother as a good source of information on solutions to breastfeeding problems. The mothers and older discussants felt that someone who had experience with breastfeeding could provide valuable advice to those who were having problems with breastfeeding. The rural discussants did not have easy access to health workers. Therefore, they relied more on the older population with more experience as compared with their urban dwellers.

### Knowledge on infant and young child feeding

About 50% (311/618) of the mothers had been informed about breastfeeding and the information was mostly obtained from the health workers (251/310, 81%), followed by maternal relatives, especially the mother. Friends, community health workers and the radio were also an important source of information about breastfeeding. The content of information received consisted of putting the newborn immediately to the breast after delivery, correct positioning techniques of baby when breastfeeding, exclusive breastfeeding, duration of the whole breastfeeding period and when to introduce other foods. The focus group discussions also reflected the fact that the knowledge on infant feeding practices was obtained mostly from the health workers and most of the discussants generally knew that exclusive breastfeeding was advocated for six months and thereafter other feeds could be introduced.

### Nutritional status

The majority of children (523/625, 83.7%) observed during the study period reported no illness and were of good nutritional status. Of the children seen 4.2% (25/594) had severe stunting; 1.7% (10/596) were severely underweight and 0.5% (3/594) had severe wasting.

Table 2 shows that underweight was significantly associated with mother’s education status (p = 0.047) and marital status (p = 0.037). A higher proportion of males (54/267, 20.2%) were underweight compared to females (32/253, 11.2%). No significant associations were observed between wasting on one hand and schooling and marital status on the other. Stunting was only significantly associated with schooling (p = 0.011).

**Table 2:** **Characteristics of caretakers and the children who were malnourished in the two districts (Mazabuka and Kafue) combined**

	**Stunting**			**Underweight**			**Wasting**		
**Characteristics**	**Total**	**n**	**(%)**	**Total**	**n**	**(%)**	**Total**	**n**	**(%)**
**Sex of child**									
Male	266	61	(22.9)	267	54	(20.2)	295	18	(6.1)
Female	250	42	(16.8)	253	32	(12.6)	271	13	(4.8)
p-value	0.154			0.049			0.519		
**Schooling**									
0-7years	298	74	(24.8)	303	62	(20.5)	333	23	(6.9)
>7 years	171	22	(12.9)	168	20	(11.9)	182	6	(3.3)
p-value	0.011			0.047			0.106		
**Marital status**									
Married	431	83	(19.3)	440	65	(14.8)	476	23	(4.8)
Not married	85	20	(23.5)	80	21	(26.3)	90	8	(8.9)
p-value		0.47			0.04			0.15	

### Signs and prevention of malnutrition

In the FGD, the discussants knew the signs of malnutrition and described it as “*condition where a child has swollen face, feet and abdomen*”. Generally there was good knowledge on the fact that malnutrition is caused by child not taking adequate food although no one mentioned the fact that the quality of the food played an important role in the causation of malnutrition.

Knowledge about the food groups was generally good with the majority of the mothers mentioning the three main food groups. The discussants from the rural setting though knowledgeable about the food groups could not practice giving the child different types of food as compared with the discussants from the urban setting.

When asked about prevention of malnutrition, the discussants talked about giving a lot of porridge and making sure that the child is always satisfied whilst the focus for others was on making sure that the child never got cold food. A few mentioned giving the child food like vegetables, porridge, groundnuts, local drink (*umunkoyo)*, oranges, bananas and bean soup as a good way of preventing malnutrition. The importance of giving varied diet with all required nutrient in the right proportion was not highlighted. Common foods mentioned that could be used in treating malnutrition were porridge and groundnuts, vegetables, and body building food.

### Care seeking

Several mothers mentioned that they would take their child to the clinic or hospital and get help from the nurses. ‘*Immediately you see the signs you take the child to the clinic where they can help you, they will also tell you the kind of food to give the baby.’* Only a few mothers said that they did not know what they would do if their child became malnourished.

## Discussion

### Initiation of breastfeeding

Although it was reported that prelacteal feeds were given to the newborn infants, all the mothers initiated breastfeeding at birth. This compares well with the results from the 2002 and 2007 ZDHS and other studies within the region [1,4,15]. Not all the mothers were aware of the protective nature of the first milk (colostrum) and tended to discard it citing reasons such as milk being ‘bad’. This practice was also observed in another study conducted in Tanzania [16]. Infants given colostrum and breastfeeding have fewer episodes of diarrhoea compared to those that are not [12]. Lack of knowledge on the advantages of giving newborn colostrum can lead to mothers discarding the first milk.

### Exclusive breastfeeding

In this study, a large proportion of infants less than four months of age, were reported to have been exclusively breastfed. In the 2002 ZDHS and a report on assessment of infant feeding practices conducted by Norwegian Programme for Development, Research and Education (NFNC) in Zambia in 2003, fewer children (less than 50%) in Mazabuka and in Kafue were exclusively breastfed [17].

Most of the caregivers knew about exclusive breastfeeding up to six months of age and were generally knowledgeable about what and when to introduce other foods to the infants. Despite this high level of knowledge about exclusive breastfeeding for up to six months, it did not translate into the communities actually putting this into practice. In both communities the rates of exclusive breastfeeding up to six months was low. The majority of those who did not exclusively breastfeed for up to six months provided some water to the infant early in the child s life. This concurs with the findings of the ZDHS reports [1,4]. In other studies conducted in the African sub-Saharan region it was reported that water alone or mixed with sugar were offered even to newborn infants and ‘light’ porridge was also offered to infants as young as two months [11,18].

### Complementary feeding

During focus group discussions, some discussants had a perception that introducing the baby to solid feeds needed to be preceded by a period of first feeding the baby on thin porridge made from maize meal. This meal is usually not enriched with other foods. The complementary foods commonly introduced do not meet the minimum standard of recommended IYCF practices with respect to food diversity, frequency of feeds and consumption of breast milk or other milk or milk products. Only 37% of youngest children aged six-twenty three months were fed in accordance to IYCF practice minimum standard [4]. This study corroborates other studies that showed that the feeds introduced are not adequate in nutrition and tend to be low in energy and micronutrients such as vitamin A, iron, zinc and other essential nutrients [19-22]. Compliance with recommended IYCF practices increases with mother’s level of education. Forty-eight percent of children whose mothers attended secondary school were fed according to the recommended IYCF practices, compared with 32% of children whose mothers had no education [4]. In other studies, education was found to improve adherence to infant feeding recommendations and reduced childhood morbidity [11,12,23-26]. National reports showed the mean day time feeds in the country to be seven but the mean night time feeds to be about five [4]. A study conducted in Ghana, reported a high frequency (21 times in a day) of breastfeeding, but feeds were of a shorter duration [27].

### Continued breastfeeding

The median age at which young children stopped breastfeeding was 18 months which was slightly less than the national median age of 20 months reported in 2002 [4]. The DHS from different countries in the sub-Saharan region showed the median duration for breastfeeding to range from 18 to 25 months [18].

### Support for breastfeeding

Fathers were mentioned to be important in supporting the mothers financially and morally to breastfeed optimally. They ensured provision of appropriate foods especially during the weaning period. Nurses were identified as important source of support for mothers or caretakers when they encountered difficulties pertaining to breastfeeding. Nurses provided most information about infant feeding practices to caregiver during the antenatal care (ANC) services and U5C visits to expectant mothers. Information included knowledge on types of food to be introduced at six months.

### Nutritional status

Most of the children’s nutritional status was normal with a lower rate of severely stunted, severely underweight and severely wasted as compared to national figures. In this study malnutrition was significantly associated with the level of education of the caretaker, which corroborates other studies in Africa that showed the higher the level of education the lower the prevalence of malnutrition [28-30]. In the 2007 ZDHS, education and wealth were inversely related to stunting, wasting and underweight.

Data showed that children born to mothers with primary level education were more likely to be stunted (49%) than children born to mothers with more than secondary education (21%), underscoring the importance of female education. Early introduction to complementary feeding was associated with a lower weight for age and increased risk of respiratory infection in a study done Malawi [25]. In this study the caregiver in the community knew about the recommended feeding practices, but this knowledge did not translate into good practice. Adherence to the recommended feeding practices by the children caretakers has beneficial effect on the growth of the infant and young child [31].

### Limitations

Funds did not permit other types of sampling especially in Mazabuka where most of the rural population was scattered over a large geographical area.

## Conclusion

The caregiver in the communities knew about the recommended feeding practices, but this knowledge did not translate into good practice. Knowing that most of the mothers will breastfeed and have heard about appropriate breastfeeding practice, is important in the development of sustainable strategies required to improve feeding practices and thus nutritional status of children.

## Abbreviations

AIDS: Acquired immune deficiency syndrome
ANC: Antenatal care
DHMT: District Health Management Team
DHS: Demographic and health survey
EBF: Exclusive breastfeeding
FANC: Focused antenatal care
FGD: Focused group discussions
HIV: Human Immunodeficiency Virus
IYC: Infants and young children
IYCF: Infant and young child feeding
MDG 4: Millennium development goal 4
NFNC: Norwegian programme for development, research and education
U5C: Under five clinic
UNICEF: United Nations Children’s Fund
WHO: World Health Organization
ZDHS: Zambia demographic and health survey
